# Utilizing the novel peroral choledochoscope for the treatment of hepatolithiasis about primary sclerosing cholangitis

**DOI:** 10.1055/a-2885-8233

**Published:** 2026-06-12

**Authors:** Xiaokui Qiu, Ximin Lin, Yulin Li, Zhongyin Zhong, Jincui Wang, Huan Peng, Zhongming Dai

**Affiliations:** 1 Department of GastroenterologyShenzhen University of Advanced Technology General HospitalShenzhenGuangdongChina

Primary sclerosing cholangitis (PSC) is a chronic immune-mediated disease. 1​ PSC patients frequently present with hepatolithiasis (HL) and the failure rate of endoscopic therapy for HL in PSC remains substantial. 2​ We report the use of a novel peroral choledochoscope (the EyeMax direct visualization system, Micro-Tech, China) for treating HL in PSC.

A 55-year-old man was admitted with a 1-month history of abdominal pain. He was diagnosed with PSC 3 years ago and had received no treatment. One month ago, HL was found in his left intrahepatic bile ducts in other hospital. He underwent endoscopic retrograde cholangiopancreatography (ERCP) with stone extraction but subsequently experienced recurrent abdominal pain. After admission, evaluation revealed a total bilirubin of 138 µmol/L and a direct bilirubin of 132 µmol/L. Magnetic resonance imaging (MRI; Fig. 1 ) showed dilatation of hilar bile duct and left intrahepatic bile ducts. With the patient’s consent, an exploratory procedure using the EyeMax system was performed. Endoscopic ultrasonography revealed bile duct wall thickening ( Fig. 2 ). During the procedure, cannulation of the distal common bile duct was achieved with a 0.025-inch guidewire-assisted sphincterotome (Boston Scientific, USA). The EyeMax system was inserted, revealing extensive fibrotic and hardened bile duct walls, with scattered dark brown stones in the hilar bile duct and left intrahepatic ducts ( Fig. 3 ). A balloon (Olympus, Japan) was positioned above the left hepatolithiasis. Following the expansion of the balloon, it was retracted toward the duodenum, thereby facilitating the passage of the stone into the duodenum ( Fig. 4 ). Repeated exploration confirmed no residual stones. The patient’s abdominal pain resolved and total bilirubin normalized after adding ursodeoxycholic acid and prednisone. Six months later, his MRI detected no stones and the liver function was normal ( Video 1 ).

**Fig. 1:**
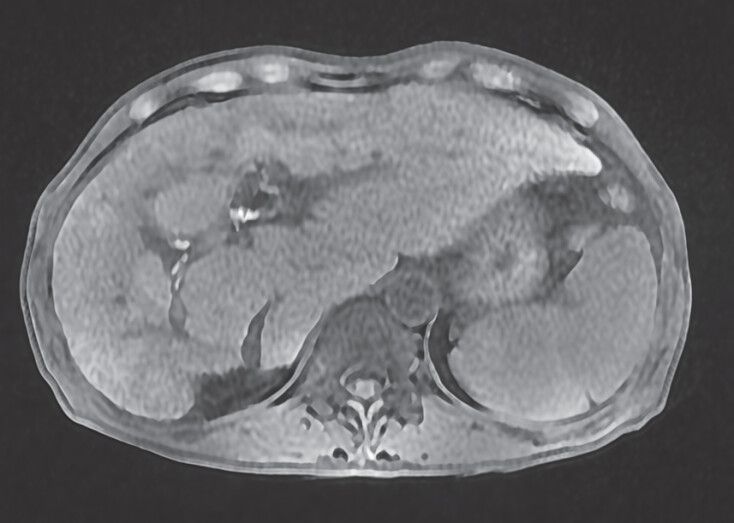
A 55-year-old man with untreated PSC experienced recurrent abdominal pain after ERCP with stone extraction, and his MRI revealed dilatation of the hilar bile duct and left intrahepatic bile ducts.

**Fig. 2:**
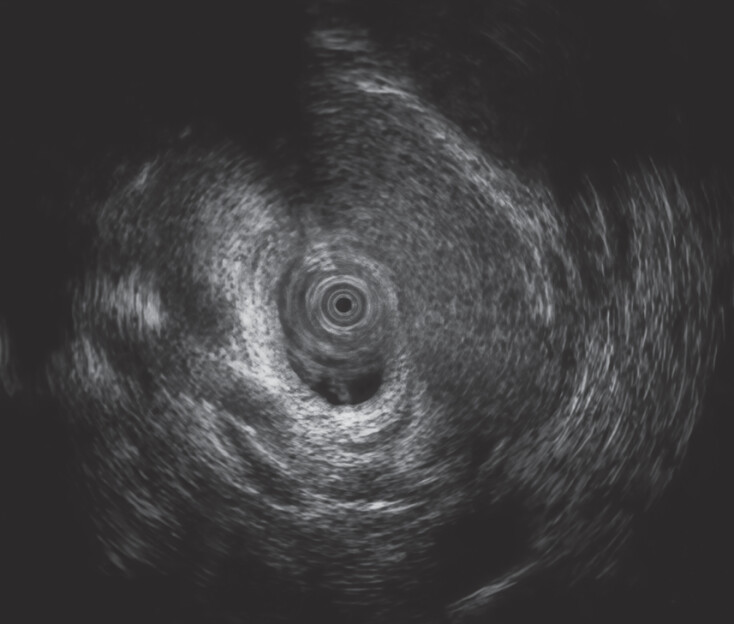
Endoscopic ultrasonography revealed bile duct wall thickening.

**Fig. 3:**
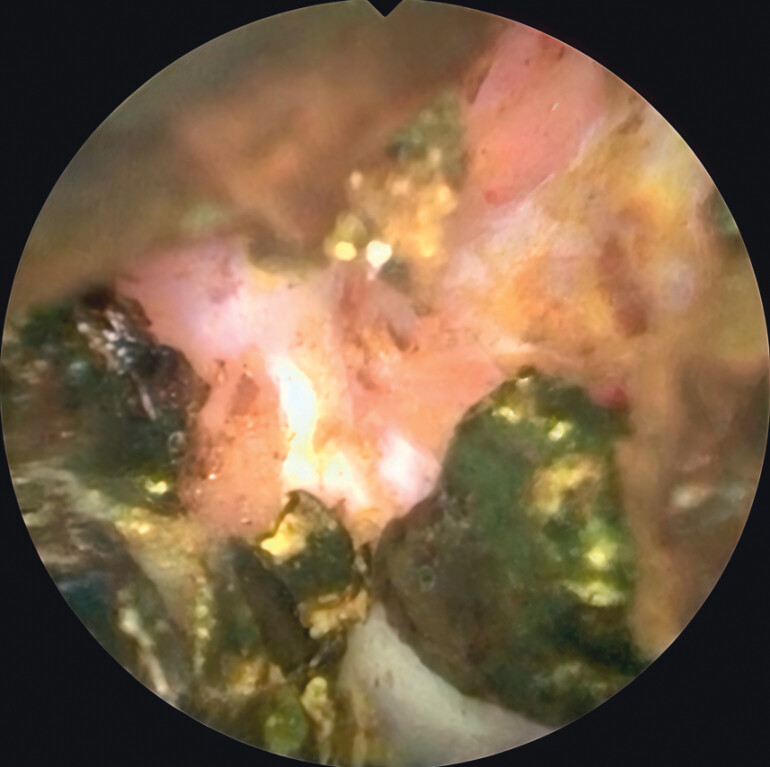
The EyeMax system was inserted, revealing extensive fibrotic and hardened bile duct walls, with scattered dark brown stones in the hilar and left intrahepatic ducts.

**Fig. 4:**
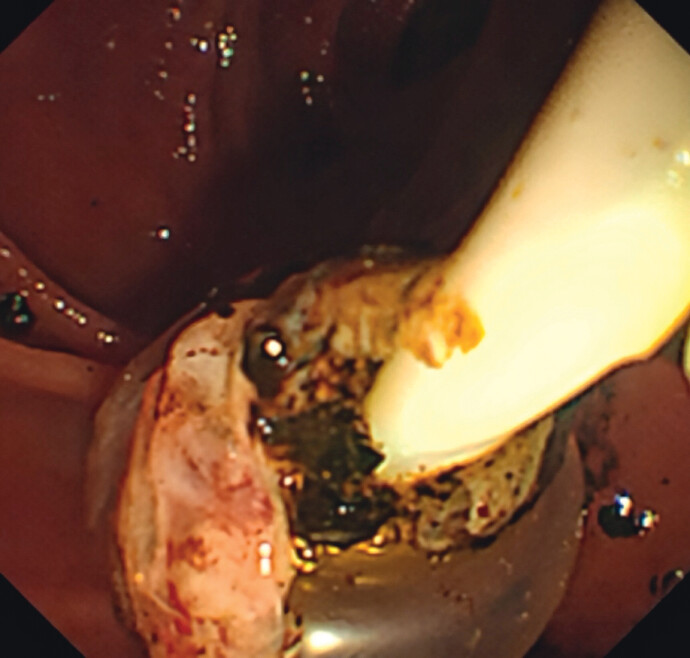
Following the expansion of the balloon, it was retracted toward the duodenum, thereby facilitating the passage of the stone into the duodenum.

**Video 1** Remove hepatolithiasis in primary sclerosing cholangitis using the EyeMax choledochoscope.


PSC is a risk factor for bile duct stones. Compared with ERCP, the novel peroral choledochoscope permits the direct visualization of the bile duct and HL in PSC patients, facilitating more appropriate treatment. Moreover, immune‑modulating therapy should be actively administered alongside relief of biliary obstruction. 3​

Endoscopy_UCTN_Code_TTT_1AR_2AL

## References

[1] Chinese Society of Hepatology, Chinese Medical Association Guidelines on the diagnosis and management of primary sclerosing cholangitis (2021) Zhonghua Gan Zang Bing Za Zhi 2022 30 169 189 10.3760/cma.j.cn112138-20211109-00786 35359068 PMC12769972

[2] Jegadeesan R Navaneethan U Lourdusamy V Endoscopic Management of Bile Duct Calculi in Patients With Primary Sclerosing Cholangitis Gastrointest Endosc 2015 81 AB364 10.1016/j.gie.2015.03.1495 25085336

[3] Liu X Wang H Liu X Efficacy and safety of immune-modulating therapy for primary sclerosing cholangitis: A systematic review and meta-analysis Pharmacol Ther 2022 237 108163 10.1016/j.pharmthera.2022.108163 35271884

